# Delayed Leucoencephalopathy as a Complication after Endovascular Therapy of Intracranial Aneurysms—A Case Series

**DOI:** 10.3390/jcm12020496

**Published:** 2023-01-07

**Authors:** Eleni Bakola, Georgia Papagiannopoulou, Lina Palaiodimou, Konstantinos Lagios, Eftychios Archontakis, Aikaterini Theodorou, Aristeidis H. Katsanos, Sokratis Triantafyllou, Vasiliki Zouvelou, Stefanos Lachanis, Dimitrios Tzanetakos, John S. Tzartos, Sotirios Giannopoulos, Georgios Tsivgoulis

**Affiliations:** 1 Second Department of Neurology, School of Medicine, National and Kapodistrian University of Athens, “Attikon” University Hospital, 12462 Athens, Greece; 2 Interventional Radiology and Neuroradiology Department, 251 Air Force Hospital, 11525 Athens, Greece; 3 Interventional Radiology Department, Red Cross Hospital, 11526 Athens, Greece; 4 Division of Neurology, McMaster University/Population Health Research Institute, Hamilton, ON L8S 4L8, Canada; 5 First Department of Neurology, National and Kapodistrian University of Athens, Aeginition Hospital, 11528 Athens, Greece; 6 Iatropolis Magnetic Resonance Diagnostic Centre, 15231 Athens, Greece; 7 Department of Neurology, The University of Tennessee Health Science Center, Memphis, TN 38163, USA

**Keywords:** enhancing white matter lesions, endovascular treatment, intracranial aneurysm, immune reaction, case series

## Abstract

We describe the clinical presentation, radiological findings, treatment and outcomes of three patients with delayed leukoencephalopathy occurring after endovascular treatment (EVT) for cerebral aneurysms—a rare, albeit recurring, complication. The symptoms occurred 6 to 12 months following the EVT of the cerebral aneurysm. Characteristic imaging findings included high-signal changes on T2 images in the white matter without diffusion restriction predominantly at the distribution of the vascular territory of the catheterized arteries, coupled with patchy gadolinium enhancement or low susceptibility weighted imaging (SWI) signals within the white-matter lesions. Steroid pulse therapy is the treatment of choice and promptly improves clinical and imaging findings. Tapering or cessation of steroids may result in clinical and imaging relapses; close- and long-term follow-up for patients presenting this complication is warranted.

## 1. Introduction

Endovascular treatment (EVT), performed with coils, stents, and flow diverters, is considered an evolving therapy for cerebral aneurysms [1]. The most common complications after EVT are hemorrhagic and thromboembolic events [2]. However, in recent years a new type of complication has been increasingly recognized: a foreign-body reaction caused by the shedding of the hydrophilic coating used on microcatheters into the blood stream and subsequent embolization in the brain parenchyma, resulting in an immune-mediated encephalopathy that may be challenging to diagnose and treat [3].

**Figure 1:**
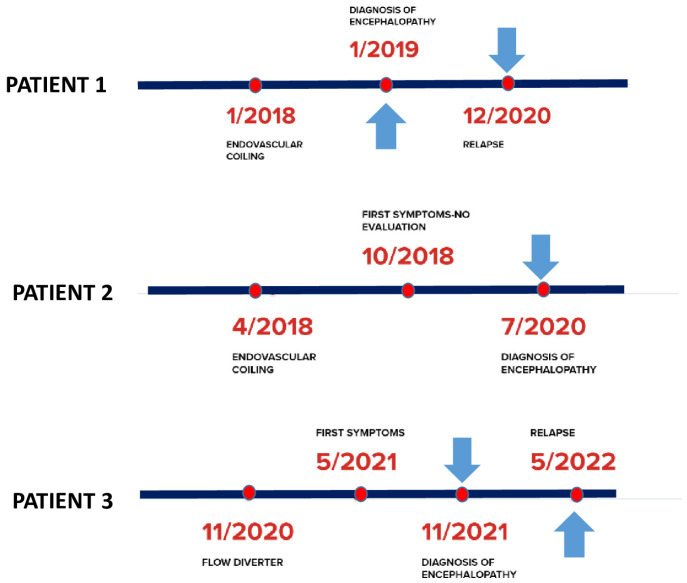
Timeline overview of three cases. Arrows suggest steroid treatment.

In view of these considerations, we describe three consecutive cases presenting at our tertiary care stroke center with delayed non ischemic cerebral enhancing (NICE) lesions after EVT of cerebral aneurysms during the last three years (Table 1, Figure 1). We also discuss the relevant literature, the clinical and imaging characteristics as well as potential treatment strategies. All patients gave informed consent to publish their data.

## 2. Case Descriptions

### 2.1. Case 1

**Figure 2:**
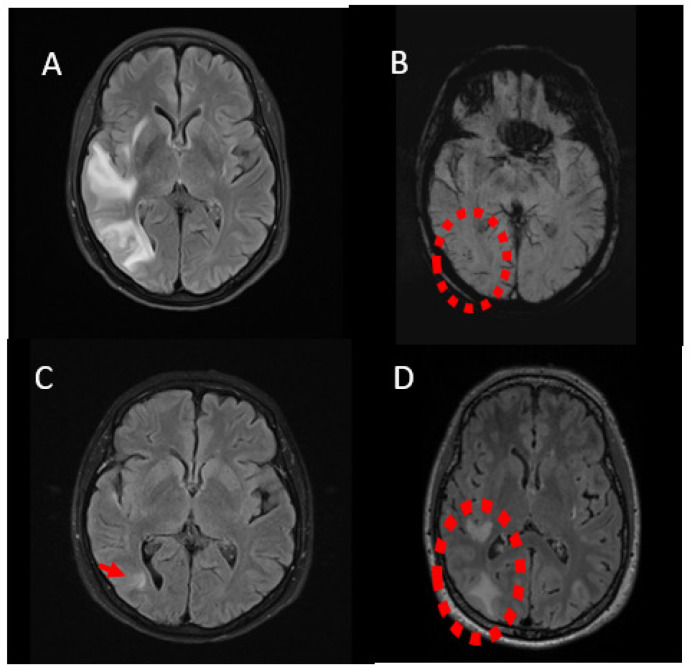
Imaging findings of case 1. Axial fluid-attenuated inversion recovery (FLAIR) sequence demonstrating white-matter hyperintensities right temporoparietal (**A**); Low-signal spots in axial SWI ((**B**); dotted circle). Follow-up MRI one month later with substantial resolution of the white matter hyperintensities in axial FLAIR sequence ((**C**); arrow); Recurrence of white-matter hyperintensities on axial FLAIR image two years later ((**D**); dotted circle).

A 64-year-old woman presented to the emergency department with subacute-onset headache, auditory hallucinations, paresthesias and weakness of the left upper limb with an initial National Institutes of Health Stroke Scale (NIHSS) score of 3 points. Her past medical history included non-traumatic subarachnoid hemorrhage (SAH) one year ago, due to aneurysm rupture of the right posterior communicating artery (PCom), which was uneventfully treated with endovascular coil embolization (Helix QC-2-4-3D, QC-3-4, QC-6-15-3D; microcatheter Marksmann FA-55150-1030). Computed tomography (CT) of the brain revealed right temporoparietal hypodense lesions. Magnetic resonance imaging (MRI) of the brain confirmed right temporoparietal subcortical lesions, surrounded by vasogenic edema without diffusion restriction. Additionally, susceptibility-weighted imaging (SWI) showed low-signal spots within the lesions, suggesting deposition of paramagnetic material and slight gadolinium enhancement in the post-gadolinium T1-weighted sequences (Figure 2A,B). Extensive work-up for infectious, paraneoplastic and autoimmune causes was negative. Based on the recent history of EVT and the MR characteristics of the lesions, the diagnosis of delayed immune-mediated encephalopathy was highly considered. Steroid pulse therapy (1000 mg of methylprednisolone iv/day for 5 days) was initiated with immediate clinical improvement (NIHSS score 0). Follow-up MRI of the brain at 30 days demonstrated significant resolution of the white matter lesions (Figure 2C).

Two years after a slow attempt in tapering steroid treatment, the patient presented with a clinical relapse with headache, dysarthria and left upper arm weakness (NIHSS score 2). Brain MRI also revealed a radiological relapse with enhancing lesions and low-signal spots on SWI along with extensive vasogenic edema, and a second steroid pulse treatment was initiated resulting in clinical and radiological recession (Figure 2D).

### 2.2. Case 2

**Figure 3:**
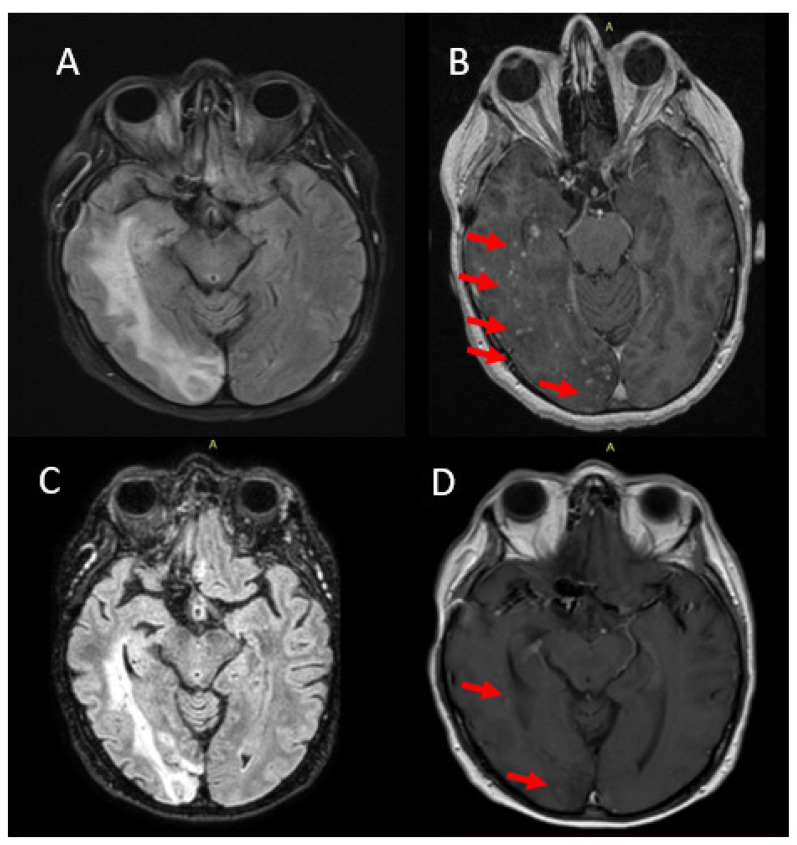
Imaging findings of case 2. White-matter hyperintensities right parietoccipital in axial FLAIR sequence ((**A**); arrows); Gadolinium-enhancement of the lesions in axial T1-weighted image ((**B**); arrows). Follow-up MRI one year later demonstrating slight vasogenic edema remission on axial FLAIR sequence (**C**); Significant improvement in axial T1-weighted image showing only slight Gd enhancement ((**D**); arrows).

A 62-year-old woman presented in our outpatient clinic with gradual left-sided hemiparesis, auditory hallucinations and seizures over the last six months (NIHSS score 3). Her medical history included non-traumatic SAH three years ago due to aneurysm rupture of the right posterior communicating artery (PCom), successfully treated with EVT and a surgical aneurysmal clipping of unruptured aneurysm in the anterior communicating artery (Acom). Six months after the EVT, the patient developed left homonymous hemianopsia and brain CT revealed right occipital hypodense lesion. Unfortunately, the patient declined further evaluation at that time. Brain MRI at the time of admission in our department revealed right occipitoparietal and occipitotemporal white matter lesions with radiological characteristics of vasogenic edema. SWI showed low-signal spots within the lesions, while post-gadolinium T1-weighted sequences demonstrated slight gadolinium-enhancement of the lesions (Figure 3A,B). Blood and cerebral spinal fluid (CSF) laboratory examinations were negative for electrolytic, infectious, neoplastic and autoimmune disorders. The patient was treated with steroid pulse therapy followed by per os steroids with significant clinical improvement (NIHSS score 0). Follow-up MRI 2 months later confirmed a significant resolution regarding the extent and the enhancement of white-matter lesions. The patient continued with oral administration of steroids and radiological improvement of the MRI white-matter lesions were preserved in follow-up MRI one year later (Figure 3C,D).

### 2.3. Case 3

**Figure 4:**
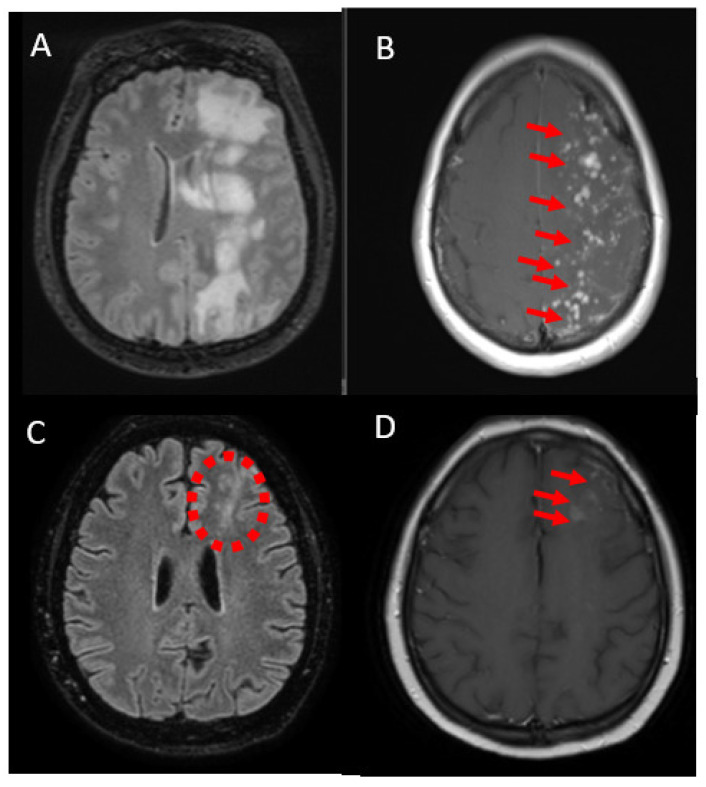
Imaging findings of case 3. FLAIR image with white matter lesions in the left hemisphere (**A**); Axial T1-weighted image showing intense Gd-enhancement ((**B**); arrows). Both axial FLAIR- sequence ((**C**); dotted circle) and axial T1-weighted image ((**D**); arrows) show radiological improvement one month later.

A 49-year-old woman was referred to our neurologic department due to gait disorders, right-sided hemiparesis, partial seizures of the right upper limb and mild cognitive and concentration difficulties, gradually deteriorating over the last 6 months (NIHSS score 4). A remarkable piece of information from her medical history was the application of a flow diverter stent (P 64-MW-HPC-450-15 FD) in a non-ruptured aneurysm of the supraclinoid segment of left internal cerebral artery (ICA) one year ago. The microcatheter that was used, had a hydrophilic coat (Rebar 18 105-5081-153). The patient was admitted to another hospital 5 months ago and brain MRI revealed multiple white-mater lesions in the left frontoparietal region with parenchymal enhancement as well as leptomeningeal enhancement after intravenous contrast administration (Figure 4A,B). A thorough diagnostic work-up including CSF analysis was negative. The patient underwent brain biopsy. The neuropathological findings were consistent with chronic necrotizing granulomatous inflammation, yet without any infectious or malignant source. Based on the previous findings, encephalopathy associated with immune reaction against coiling was a plausible diagnosis and the patient was treated with steroid pulse therapy continued by per os steroids with significant symptomatic recession (NIHSS score 1). A follow-up MRI brain one month later confirmed a significant reduction of white-matter lesions (Figure 4C,D). Tapering of corticosteroids resulted in clinical and neuroimaging recurrence of the immune-mediated leukoencephalopathy six months later with an NIHSS score of two that was treated with steroid pulse therapy.

## 3. Discussion

Over the last few years, delayed non ischemic cerebral enhancing lesions suggesting immune-mediated leukoencephalopathy have been recognized as an increasing complication after aneurysm EVT, including coils, stents, and flow diverters with an estimated incidence between 0.05% and 2.3% mostly observed in women aged 41–65 years with median time of weeks up to 12 months after the procedure [3,5,6,7,8]. Consistently, in the above-described cases all patients were women between 49 and 64 years old and the symptoms occurred 6 to 12 months following the EVT of the cerebral aneurysm. These 3 cases occurred out of 226 cases with ruptured and unruptured cerebral aneurysms that were evaluated since 2018 at our Neurology Department.

Brain MRIs of all similar cases reported so far in the literature have distinctive features: T2-fluid-attenuated inversion recovery hyperintensities predominantly in the subcortical white matter and at the grey–white matter border, predominantly at the distribution of the vascular territory of the catheterized arteries as well as perilesional edema, which varies in extent. Gadolinium enhancement is either solid or rim-shaped. Partially, low signals in SWI in the fluid-attenuated inversion recovery (FLAIR) hyperintensities are reported, probably due to deposition of microembolic paramagnetic coil-related materials [3,4,5,6,7,8,9,10,11,12,13,14,15,16,17].

Brain biopsy was performed in few cases demonstrating granulomas, angiitis, periadventitial foreign-body giant cell response, and microabscesses encasing foreign material, neutrophilic granulocytes, and multinucleated macrophages [5,9,11]. Brain biopsy may be useful to atypical cases not properly responding to treatment in order to verify the clinical and radiological entity. The presumed underlying pathophysiological mechanism includes granulomatous reaction caused by foreign body emboli from the hydrophilic coating of EVT devices, contrast-induced encephalopathy and nickel or bioactive polyglycolic/polylactic acid coil sensitivity [3]. Pulsatile blood flow in the cerebral artery may contribute to continuous mechanical scraping of coil filaments within intracranial aneurysms, resulting in their dislodgment and embolization in distal arterial branches [11].

In terms of treatment, steroid pulse therapy followed by oral prednisone is considered the first treatment option, leading to significant resolution of neurological symptoms along with improvement of MR findings [3,4,5,6,9,10,14,15,16]. Nevertheless, the lesions seem to have steroid dependency, as during tapering or discontinuation of the steroid treatment, clinical and neuroimaging relapses are observed requiring reinitiation or dose increase [4,5,9,10]. Eventually, in such cases, prolonged immunosuppressive therapy may be considered [7]. Apart from clinical examination and evaluation of new neurological symptoms, clinicians should monitor closely for post EVT radiologic parenchymal edematous changes in follow-up brain MRIs, suggesting immune-mediated leukoencephalopathy in order to initiate immunological treatment swiftly and effectively [17].

In conclusion, delayed leucencephalopathy is a rare complication after EVT treatment of cerebral aneurysms. Approximately a hundred cases have been published so far over the last 14 years (Table 2). The main reason for discussing and presenting these three cases is to contribute more evidence to the relevant literature, to provide information about the stents and coils that have been used, discuss their course, treatment and outcome. Most of the cases were treated successfully with corticosteroids. Few of them required other immunosuppressive therapy based on individual decisions by taking into consideration the clinical course and the effects of the MRI lesions. Therefore, firm conclusions or recommendations regarding the treatment options cannot be made. However, in the future, based on the growing awareness and evidence of this disorder more concrete treatments strategies could be determined.

Nevertheless, it is remarkable that in the three largest cohort studies [3,6,7] 16, 7 and 5 patients, respectively, have been identified with delayed encephalopathy after EVT treatment in a time period of 12 years. In our center, three cases have been presented during the last 3 years. This discrepancy between the above-mentioned cohort studies and our experience may be interpreted as following: when the first case presents and is diagnosed in a comprehensive stroke center, the awareness of this diagnosis for future cases is high and this leads to the earlier recognition of this disorder. Furthermore, it can be assumed that the exponential increase in the use of endovascular coils, stents and other devices may eventually have an impact on the incidence of delayed encephalopathy. Finally, the fact that EVT has been established as the current, first-line therapy for cerebral aneurysms over microsurgical clipping may also have an impact on the incidence of delayed encephalopathy.

## Figures and Tables

**Table 1:** Overview of described cases with delayed white matter lesions complicating endovascular therapy for intracranial aneurysms.

Scheme	EVT Procedure/ Parent Vessel	Interval between Procedure and Leucoencephalopathy	Symptoms	MRI Findings	Biopsy	Treatment/ Response	Follow Up Duration
Case 1 * F/64	Endovascular coiling/ right PCom	12 months	headache, auditory hallucinations, paresthesias and weakness of the left upper limb	Enhancing lesions, vasogenic edema, low SWI signals	No	Steroids **/Initial improvement, recurrence after tapering	3.5 years
Case 2 F/62	Endovascular coiling/ right PCom	6 months	left homonymous hemianopsia, left-sided hemiparesis, auditory hallucinations and seizures	Enhancing lesions, vasogenic edema, low SWI signals	No	Steroids **/improvement	3 years
Case 3 F/49	Flow diverter stent/ supraclinoid segment of left ICA	6 months	gait disorders, right-sided hemiparesis, partial seizures of the right upper limb, mild cognitive impairment	Enhancing lesions, vasogenic edema, low SWI signals	Yes-chronic necrotizing granulomatous inflammation	Steroids **/improvement, recurrence after tapering	1 year

* Case 1 has been previously published [4].** 1000 mg of methylprednisolone iv/day for 5 days followed by oral prednisolone 1mg/kg with gradual tapering.

**Table 2:** Studies presenting NICE lesions after EVT of aneurysms.

Authors, Year	Type of Study	Patients (Sex/Age)
Ikemura et al., 2020 [3]	Cohort study	16 out of 1722 procedures F 71.3%, mean age 59
Shotar et al., 2016 [5]	Case series	2 out of 374 patients, M/45, F/54
Nakagawa et al., 2020 [6]	Cohort study	7 patients out of 305 F 100%-mean age 59
Bayas et al., 2022 [7]	Cohort study	5 out of 746 patients, F 100%, mean age 51
Shotar et al., 2022 [8]	Cohort study	31 out of 58815 procedures 84% F, mean age 45
Shapiro et al., 2015 [9]	Case series	5 patients
Grewal et al., 2015 [10]	Case report	F/65
Fealy et al., 2008 [11]	Case report	F/58
Skolarus et al., 2010 [12]	Case report	F/46, F/56
Ulus et al., 2012 [13]	Case report	F/41
Cruz et al., 2014 [14]	Case series	7 patients, F 86%, mean age 54
Lobotesis et al., 2015 [15]	Case report	F/60
Park et al., 2018 [16]	Case report	F/64, F/52
Bakola et al., 2021 [4]	Case report	F/64
Ridwan et al., 2021 [17]	Systematic review and case report	F/53

## Data Availability

The datasets used and analysed during the current study are included in this article. More detailed datasets are available from the corresponding author on reasonable request.
